# Crystal and Electronic Structure of Ba_12_Ga_15_As_22_

**DOI:** 10.1063/4.0001126

**Published:** 2025-10-27

**Authors:** Spencer Watts, Larissa Najera, Sviatoslav Baranets

**Affiliations:** Louisiana State University, Baton Rouge, Louisiana, USA

## Abstract

The novel ternary Zintl phase Ba12Ga15As22 was synthesized and structurally characterized. Structural data collected via single-crystal X-ray diffraction methods revealed the presence of homoatomic [Ga2]4+ bonds embedded within chains of edge- and corner-sharing [GaAs4] tetrahedra. Synchrotron powder diffraction data, processed via Rietveld refinement, further confirm the structural model. Ba12Ga15As22 crystallizes in the monoclinic crystal system with space group P21/c (a = 13.3334(10), b = 20.6392(16), c = 20.0336(15), β = 108.7168(14)) and adopts a previously unreported structure type. Ba12Ga15As22 is heavily disorder and its charge-balance can be described using the Zintl concept as follows: Ba12Ga15As22 = (Ba2+)12(Ga3+)12([Ga2]4+)1.5(As3–)22. Electronic structure calculations were performed to further investigate the bonding and electronic properties, motivated by expectation of semiconducting properties and suitability for thermoelectric applications.

Ba12Ga15As22 was found to exhibit a theoretical band gap of approximately 0.61 eV.

